# Epigenetic Regulation of *BMP2* by 1,25-dihydroxyvitamin D_3_ through DNA Methylation and Histone Modification

**DOI:** 10.1371/journal.pone.0061423

**Published:** 2013-04-19

**Authors:** Baisheng Fu, Hongwei Wang, Jinhua Wang, Ivana Barouhas, Wanqing Liu, Adam Shuboy, David A. Bushinsky, Dongsheng Zhou, Murray J. Favus

**Affiliations:** 1 Department of Orthopedic Surgery, Provincial Hospital Affiliated to Shandong University, Jinan, Shandong, People’s Republic of China; 2 Section of Endocrinology, University of Chicago Pritzker School of Medicine, Chicago, Illinois, United States of America; 3 Department of Medicinal Chemistry & Molecular Pharmacology, College of Pharmacy, Purdue University, West Lafayette, Indiana, United States of America; 4 Division of Nephrology, University of Rochester School of Medicine and Dentistry, Rochester, New York, United States of America; Albany Medical College, United States of America

## Abstract

Genetic hypercalciuric stone-forming (GHS) rats have increased intestinal Ca absorption, decreased renal tubule Ca reabsorption and low bone mass, all of which are mediated at least in part by elevated tissue levels of the vitamin D receptor (VDR). Both 1,25-dihydroxyvitamin D_3_ (1,25(OH)_2_D_3_) and bone morphogenetic protein 2 (BMP2) are critical for normal maintenance of bone metabolism and bone formation, respectively. The complex nature of bone cell regulation suggests a potential interaction of these two important regulators in GHS rats. In the present study, *BMP2* expression is suppressed by the VDR-1,25(OH)_2_D_3_ complex in Bone Marrow Stromal Cells (BMSCs) from GHS and SD rat and in UMR-106 cell line. We used chromatin immunoprecipitation (ChIP) assays to identify VDR binding to only one of several potential binding sites within the *BMP2* promoter regions. This negative region also mediates suppressor reporter gene activity. The molecular mechanisms underlying the down-regulation of *BMP2* by 1,25(OH)_2_D_3_ were studied in vitro in BMSCs and UMR-106 cells using the DNA methyltransferase inhibitor 5-aza-2′-deoxycytidine (DAC) and the histone deacetylase inhibitor trichostatin A (TSA). Both DAC and TSA activate *BMP2* expression in combination with 1,25(OH)_2_D_3_. Bisulfite DNA pyrosequencing reveals 1,25(OH)_2_D_3_ to completely hypermethylate a single CpG site in the same *BMP2* promoter region identified by the ChIP and reporter gene assays. ChIP assays also show that 1,25(OH)_2_D_3_ can increase the repressive histone mark H3K9me2 and reduce the acetylation of histone H3 at the same *BMP2* promoter region. Taken together, our results indicate that 1,25(OH)_2_D_3_ binding to VDR down-regulates *BMP2* gene expression in BMSCs and osteoblast-like UMR-106 cells by binding to the *BMP2* promoter region. The mechanism of this 1,25(OH)_2_D_3_-induced transcriptional repression of *BMP2* involves DNA methylation and histone modification. The study provides novel evidence that 1,25(OH)_2_D_3_ represses bone formation through down-regulating *BMP2* expression both in vivo and in vitro.

## Introduction

Hypercalciuria in both Genetic Hypercalciuric Stone-forming (GHS) rats and patients with idiopathic hypercalciuria (IH), arises from intestinal Ca hyperabsorption, increased bone resorption, and decreased renal tubule Ca reabsorption [1]–[4]. As a result, the urine is supersaturated with respect to calcium and oxalate and calcium phosphate and therefore is predisposed to kidney stones of calcium salts. Low bone mass is also found in both GHS rats [5] and patients with IH [6], [7], but the pathogenesis of the bone disease is incompletely known. In previous studies, we have reported high levels of VDR in GHS rat intestine, kidney and BMSCs, which can account for all of the changes in calcium metabolism that lead to hypercalciuria [8]–[11]. *BMP2* is essential for bone formation [12], and BMP2 gene expression is lower in bone cells, kidney and intestine of GHS rats. Thus, we hypothesize that VDR down-regulates *BMP2* expression which in turn contributes to low bone mass in GHS rats.

1,25(OH)_2_D_3_, the biologically active metabolite of vitamin D, regulates gene expression in many tissues via binding to its corresponding intra-nuclear receptor, VDR, a member of the steroid hormone receptor superfamily [13]. 1,25(OH)_2_D_3_ functions in a variety of biological processes such as calcium homeostasis, cell proliferation and cell differentiation [14]. In particular, it is a critical regulator of bone turn-over [15], [16]. It is widely accepted that vitamin D is vital to bone growth, as its deficiency can result in rickets and osteomalacia [17]. Previous studies have demonstrated that 1,25(OH)_2_D_3_ increases bone mass *in vivo* [18], [19] and enhances the functional activity of mature osteoblasts *in vitro* [20]. However, the physiological significance of vitamin D is not without controversy. In VDR knockout mice, vitamin D administration reportedly down-regulated bone formation [21], [22]. *Phex*, a marker of the mature osteoblasts, and *Runx2*, a key regulator of bone formation in vivo, were down-regulated by 1,25(OH)_2_D_3_ [23]–[25]. Recent research suggests that a daily regimen of exogenous 1,25(OH)_2_D_3_ inhibits the BMP2 induced signaling pathway *in vitro,* thus effectively suppressing bone mineralization [26]. Despite the ongoing controversy, the detailed mechanisms of 1,25(OH)_2_D_3_ in bone formation have yet to be clarified.

Bone morphogenetic proteins (BMPs) belong to the transforming growth factor-β (TGF-β) superfamily and were first identified by their ability to initiate osteogenic differentiation [27], [28]. BMP2, one of the most well characterized BMPs, is an osteogenic factor that may stimulate osteoblast differentiation and osteogenic nodule formation *in vitro*, as well as bone formation *in vivo* [29], [30]. It is a key mediator of bone development and repair [31]–[33] and is required for fracture healing [34]. BMP2 plays a central role in initiating and regulating bone formation, so any factors that regulate the expression of BMP2 would be expected to influence bone formation.

Based on our current data, 1,25(OH)_2_D_3_ has both positive and negative regulatory functions in bone formation. However, the interaction between 1,25(OH)_2_D_3_ and *BMP2* is not fully known, and further studies may reveal new biologic effects of 1,25(OH)_2_D_3_ on bone formation.

In the present study, we demonstrate that *BMP2* expression is down-regulated by 1,25(OH)_2_D_3_ in both BMSCs and UMR-106 cells through DNA methylation and histone modification. Our results support the negative regulation of *BMP2* expression by 1,25(OH)_2_D_3_.

## Materials and Methods

### Animals

The colony of GHS rats was created by screening for spontaneous hypercalciuria and then selectively breeding hypercalciuric male and female Sprague-Dawley (SD) rats (Harlan, Inc., Indianapolis, IN) [2]. The hypercalciuric offspring of the breedings called GHS rats were raised at the University of Rochester and shipped to the University of Chicago at 6–7 weeks of age with the body weight in 220–250 g range. SD rats purchased from Harlan, Inc. (Indianapolis, IN) were matched for age and body weight to the GHS rats.

This study was carried out in strict accordance with the recommendations in the Guide for the Care and Use of Laboratory Animals of the National Institutes of Health. All animal experiments were approved by the University of Chicago Institutional Animal Care and Use Committee.

GHS and SD rats were sacrificed by CO_2_ narcosis followed by cervical dislocation. Tissues were removed, flash frozen in liquid nitrogen, and then subjected to rapid protein isolation and RNA extraction assays.

### Cell Culture

The BMSCs from long bones of GHS and SD rats were isolated as previously described with only minor modifications [35]. Ammonium chloride solution (STEMCELL Technologies Inc. Vancouver, Canada) was used to remove the red blood cells according to the manufacturer’s directions. The BMSCs were seeded into 6-well plates at 1×10^6^ cells/well and cultured in Dulbecco’s modified Eagle’s medium (DMEM) containing 10% fetal bovine serum (FBS), 100 U/ml penicillin, and 100 ug/ml streptomycin in a humidified 5% CO_2_ incubator at 37°C.

The UMR-106 cell line was obtained from American Type Culture Collection (Manassas, VA) and cultured in the same medium and conditions as the BMSCs. Cells were passed every 3–4 days when approaching confluence using the fresh medium and were not used beyond passage 15.

### In Vitro Treatment

Cells were seeded into 6-well plates at 1×10^6^ cells/well for 24 hr and then the media were changed. For time-response trials, BMSCs from GHS and SD rats were incubated with 1,25(OH)_2_D_3_ (Sigma, St Louis, MO) dissolved in ethanol at 10^−8 ^M for 6, 12 and 24 hr. Control cultures were administered with an equivalent volume of 100% ethanol. UMR-106 cells were incubated with 1,25(OH)_2_D_3_ at 10^−8 ^M for 1, 6, 12, 24, 36 and 48 hr for the time course experiments. For dose-response trials, cells were incubated with 1,25(OH)_2_D_3_ at 10^−7^, 10^−8^ and 10^−9^ M for 24 hr.

To determine the potential modulatory roles of DNA methylation and histone modification in *BMP2* expression, BMSCs and UMR-106 cells were cultured in the presence of DAC (Sigma, St Louis, MO) at 0.5, 1.0 and 2.0 µM for 3 days, with or without the addition of 1,25(OH)_2_D_3_ (10^−8 ^M) for an additional 24 hr. And TSA (Sigma, St Louis, MO) was used to treat at 20, 100 and 500 nM for 8 hr, with or without a subsequent addition of 1,25(OH)_2_D_3_ (10^−8 ^M) for an additional 24 hr.

### Western Blot

Nuclear extracts from BMSCs and rat tissues were isolated using the Nuclear and Cytoplasmic Extraction kit (Pierce, Inc., Rockford, IL) according to the manufacturer’s directions. Protease Inhibitor Cocktail (Sigma, St Louis, MO) was added into the reagent prior to extraction. Protein concentrations were assessed using DU® Series 500 (Beckman, Fullerton, CA). Nuclear proteins were separated by 12% SDS-PAGE gel electrophoresis when they were then transferred to nitrocellulose membranes using IBlot Gel Transfer Stacks (Invitrogen, Grand Island, NY) and blotted with anti-VDR (Enzo Life Sciences, Farmingdale, NY) and anti-β-actin antibody (Santa Cruz Biotechnology, Santa Cruz, CA). The blotted proteins were detected by an ECL Western Blotting kit (Amersham Biosciences, Piscataway, NJ).

### Total RNA Extraction and Quantitative Real-time PCR

Total RNA extraction was performed using TRIzol Reagents (Invitrogen, Grand Island, NY). cDNA synthesis was performed for 2 hr at 37°C with 1 ug of total RNA, using a High Capacity cDNA Reverse Transcription Kit (Applied Biosystems, Carlsbad, CA) according to the manufacturer’s directions. qRT-PCR was performed using SYBR green master PCR mix (Applied Biosystems, Carlsbad, CA). Gene expression of *BMP2* was normalized to that of *GAPDH*. PCR products were quantified using the comparative cycle threshold (Ct) method as described previously [36]. The primer sequences for rat BMP2 and GAPDH were as follows: *BMP2* (Forward) 5′-CCT ATA TGC TCG ACC TGT AC -3′, (Reverse) 5′-CCC ACT CAT TTC TGA AAG TTC-3′; *GAPDH* (Forward) 5′-GCA CAG TCA AGG CTG AGA AT-3′, (Reverse) 5′-TGA AGA CGC CAG TAG ACT CC -3′.

### ChIP Assay

The ChIP assay was performed as previously described with minor modifications [37]. The UMR-106 cells incubated with drugs were cross-linked by adding 270 µL 37% formaldehyde into 10 ml mediums for 10 min. The cross-link was stopped by the addition of 0.125 M glycine (final concentration). Cell lysates were sonicated to shear DNA to an average length of 200 to 1000 bps. VDR antibody (Enzo Life Sciences, Farmingdale, NY), antibody against histone H3 dimethylated lysine 9 (H3K9me2) (Millipore, Billerica, MA) and antibody against acetyl-histone H3 (Millipore, Billerica, MA) were used to immunoprecipitate the DNA-protein complexes, respectively. Normal IgG (Santa Cruz Biotechnology, Santa Cruz, CA) was used as a negative control. After an overnight incubation with the antibody, antibody/histone complexes were collected by adding 80 µL of Protein A Agarose/Salmon Sperm DNA (Millipore, Billerica, MA). The protein A agarose/antibody/chromatin complexes were then washed with the following buffers in the respective order (Millipore, Billerica, MA): low salt wash, high salt wash, LiCl wash, and TE buffer. DNA acquired before precipitation was used to assess the presence of genes following the ChIP procedure and designated “Input”. The DNA was eluted with 1% SDS and 0.1 M NaHCO3 elution buffer, and then subjected to reverse cross-linking and proteinase digestion. Ultimately, the DNA fragments were purified using QIAquick PCR Purification Kits (Qiagen, Valencia, CA) and subjected to PCR.

The entire BMP2 and its flanking regions (a total of 28,545 bps) were screened for putative VDR3-response elements (VDREs). Only the most typical DR3 type consensus sequence was screened, i.e. the direct repeat of the hexameric core sequence RGDKYR (R = G or A, D = A, G or T, K = G or T, Y = C or T). We used the online “DNA Pattern Find” program for the motif screening. Finally, eight putative VDREs were identified and corresponding primer pairs for detecting these loci were designed (Table 1).

**Table 1:** Putative VDREs regions and corresponding primers used in ChIP assays.

Region	Putative VDRE	Start	End	Primer sequences
A	CACTCCATGTGACCT	121365229	121365243	5′-TTTCCTCAGCACGTCTTCCTCACT -3′
				5′-AGCCTGCCTGAAGAAGAGTGGATT -3′
B	TGACCTACTTGCTCC	121367554	121367568	5′-CAGGGAGCACAGATCATTGGGAAT -3′
				5′-TTCGGGAGAGCAAAGAGTTGCTGA -3′
C	CGCCCCGCCCCGCCCCG	121371437	121371453	5′-ATTTGCCCTAAACTCGGGCATCTG -3′
				5′- TTCGTCCCGAGCTGCCAAT-3′
D	AGGTCACCCGGTTTG	121372034	121372048	5′- AGCACAGTCTTACCCTCAACGCT-3′
				5′- GCATCCCTCAGCCTGACTCT-3′
E	AGAGCAAATGGTGTG	121382425	121382439	5′-CCCGTGAGAGCAAATGGTGTGTTT -3′
				5′-CCTTGAGTTCTGCACGCTTTGCTT -3′
F	TACACTGTATAACCT	121384896	121384910	5′-GTGTGATGTCAGTGCCAAGCATCT -3′
				5′-CTTTGCAGAGGGAAACCCATTCCT -3′
G	TGCACTGGTCAAACT	121390091	121390105	
				5′-AGGGACCAGATTATGCACAGACCA -3′
H	AGTGTACCCAGAGTG	121390193	121390207	5′-AAATGGAGCGAATCTCTGCCTCTG -3′

The start and end nucleotide positions are based on the Rat genome assembly version Baylor 3.4/rn4. Region G and Region H share one primer pair.

### Bisulfite DNA Pyrosequencing

Genomic DNA was isolated using the Genomic DNA Isolation Kit (Qiagen, Valencia, CA) from UMR-106 cells incubated with 1,25(OH)_2_D_3_ or vehicle. About 1 µg of rat genomic DNA was treated with bisulfite conversion reagents using the MethylEasy Xceed Rapid DNA Bisulfite Modification Kit (Human Genetic Signatures, Australia) according to the manufacturer’s instruction. The bisulfite converted genomic DNA was amplified using BMP2-F1 (5′-AGTGGGGGAATTTAGAGGTAAG-3′) and BMP2-R1 (5′-TATCRTACCRACCCCTCRAAACCCC-3′) primers with the following cycling condition: 95°C 3 min, then 30 cycles of 95°C 30 sec, 56°C 30 sec and 72°C 60 sec. About 2% of the PCR products were subject to a second round of amplification using nested primers BMP2-F2 (5′-AGAGGTAAGTTTATTTTGAATTTG-3′) and BMP2-R2 (5′-CTTCRTCCCRAACTACCAATCCC-3′) with the same PCR procedural condition as above. The amplified PCR products were then subcloned; 25 clones from each ligation were randomly picked and sequenced on an Applied Biosystems 3130 instrument. Fractional DNA methylation [#C/(#C+#T)] for each CpG site was determined based on the number of times it was sequenced as a T or as a C.

### Reporter Assays

The fragment of rat *BMP2* promoter region (Region C) containing the VDR binding site was generated by PCR using primers Forward 5′-ATTTGCCCTAAACTCGGGCATCTG-3′ and Reverse 5′- TTCGTCCCGAGCTGCCAAT-3′ and was gel-purified with Gel Extraction Kit (Qiagen, Valencia, CA). The fragment was first cloned into pCR®2.1 vector using the T-A Cloning Kit (Invitrogen, Carlsbad, CA) and sequenced thereafter. The plasmids containing region C were amplified and digested with XhoI and KpnI (New England Biolabs, Ipswich, MA). The target DNA fragment was then gel-purified as before and cloned into the pGL3 Promoter Vector (Promega, madison, WI) which contains an SV40 promoter upstream of the luciferase gene. Subsequently, the constructed pSV40-BMP2 BR-Luciferase plasmid was transfected into the UMR-106 cells with Lipofectamine 2000 Reagent (Invitrogen, Carlsbad, CA) following the manufacturer’s protocol. 24 hr after transfection, the cells were treated with 10^−8 ^M 1,25(OH)_2_D_3_ for additional 24 hr and then were lysed with Tropix Lysis Buffer (Applied Biosystems, Carlsbad, CA). The luciferase and β-Galactosidase activities were measured using the Luciferase & β-Galactosidase Reporter Gene Assay System (Applied Biosystems, Carlsbad, CA) according to the manufacturer’s guidelines. β-Galactosidase activity was used to normalize transfection efficiency.

### Data Analysis

Statistical analysis was performed using the Student’s t-Test for unpaired comparisons. Data were presented as means ± SE, and p<0.05 was considered significant.

## Results

### VDR Levels and *BMP2* Expression in GHS and SD Rats

**Figure 1:**
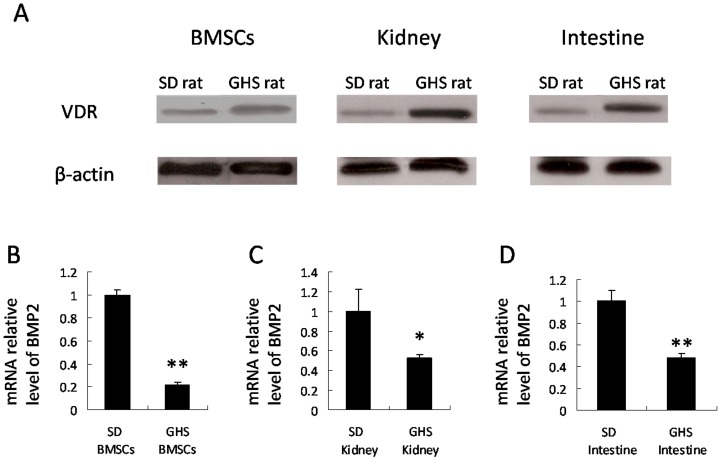
VDR levels and *BMP2* expressions in GHS and SD rats. Nuclear proteins were isolated from BMSCs and tissues of SD and GHS rats and were subjected to Western blot using anti VDR. The representative data are shown in (A). Expressions of *BMP2* in BMSCs, kidney and intestine tissues from GHS rats decrease relative to SD rats (B, C, D). (*, P<0.05; **, P<0.01).

VDR levels are higher in BMSCs from GHS rats compared to SD rats (Figure 1A). Using qRT-PCR we found BMSCs from GHS rats had a 20% lower *BMP2* mRNA expressions (P<0.01) compared to SD rats (Figure. 1B). Kidney and intestinal tissues of GHS rats had higher VDR levels and lower BMP2 mRNA expression compared to levels in the SD rat tissues (Figure. 1A and C, D).

### Effects of 1,25(OH)_2_D_3_ on *BMP2* Expression

**Figure 2:**
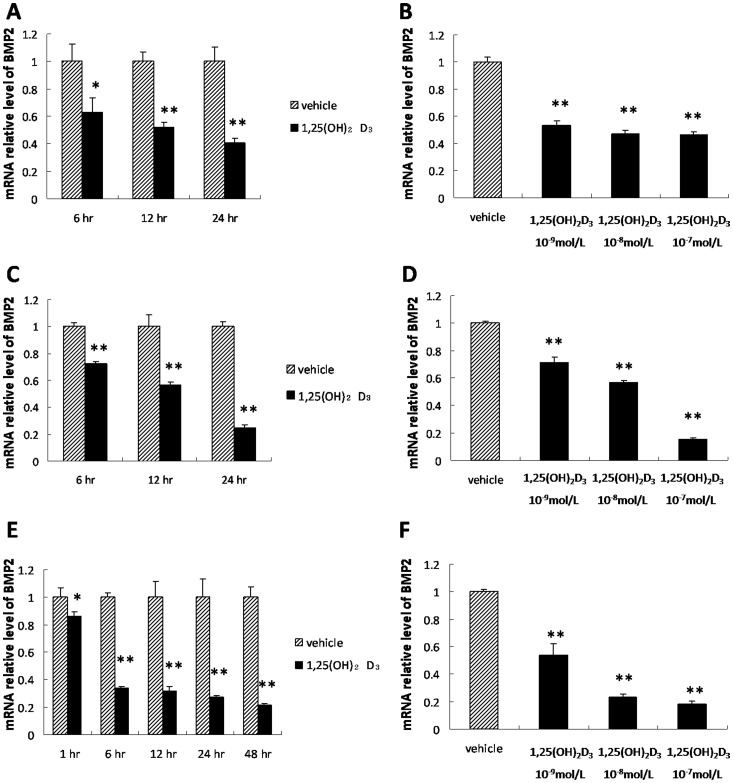
Repression of *BMP2* by 1,25(OH)_2_D_3_. A. qRT-PCR of *BMP2* expression in BMSCs from SD rats at several time points treated with 10^−8^ mol/L 1,25(OH)_2_D_3_. B. qRT-PCR of *BMP2* expression in BMSCs from SD rats treated with a range of concentrations of 1,25(OH)_2_D_3_. C. qRT-PCR of *BMP*2 expression in BMSCs from GHS rats treated with 10^−8^ mol/L 1,25(OH)_2_D_3_ at different points in time. D. qRT-PCR of *BMP2* expression in BMSCs from GHS rats treated with step-wise concentrations of 1,25(OH)_2_D_3_. E. qRT-PCR of *BMP2* expression in UMR-106 cells treated with 10^−8^ mol/L 1,25(OH)_2_D_3_ at multiple time points. F. qRT-PCR of *BMP2* expression in UMR-106 cells treated with varying concentrations of 1,25(OH)_2_D_3_. (*, P<0.05; **, P<0.01).

*BMP2* mRNA expression in BMSCs was significantly down-regulated in the presence of 1,25(OH)_2_D_3_ at 6, 12 and 24 hr in SD (Figure. 2A) and GHS rats (Figure. 2C). In BMSCs, suppression of BMP2 mRNA by 1,25(OH)_2_D_3_ was dose-dependent in both SD (Figure. 2B) and GHS rats (Figure. 2D).

Using UMR-106 cells cultured with 1,25(OH)_2_D_3_ over 1–48 hr created a time-dependent decrease in *BMP2* mRNA levels (Figure. 2E). A dose dependent down-regulation of *BMP2* expression by 1,25(OH)_2_D_3_ also occurred in the UMR-106 cells (Figure. 2F). At concentrations of 10^−9 ^M, 10^−8 ^M and 10^−7 ^M, 1,25(OH)_2_D_3_ reduced *BMP2* mRNA to 55%, 25%, and 18% of control levels, respectively (P<0.01).

### VDR Binding to Rat *BMP2* Promoter Region

**Figure 3:**
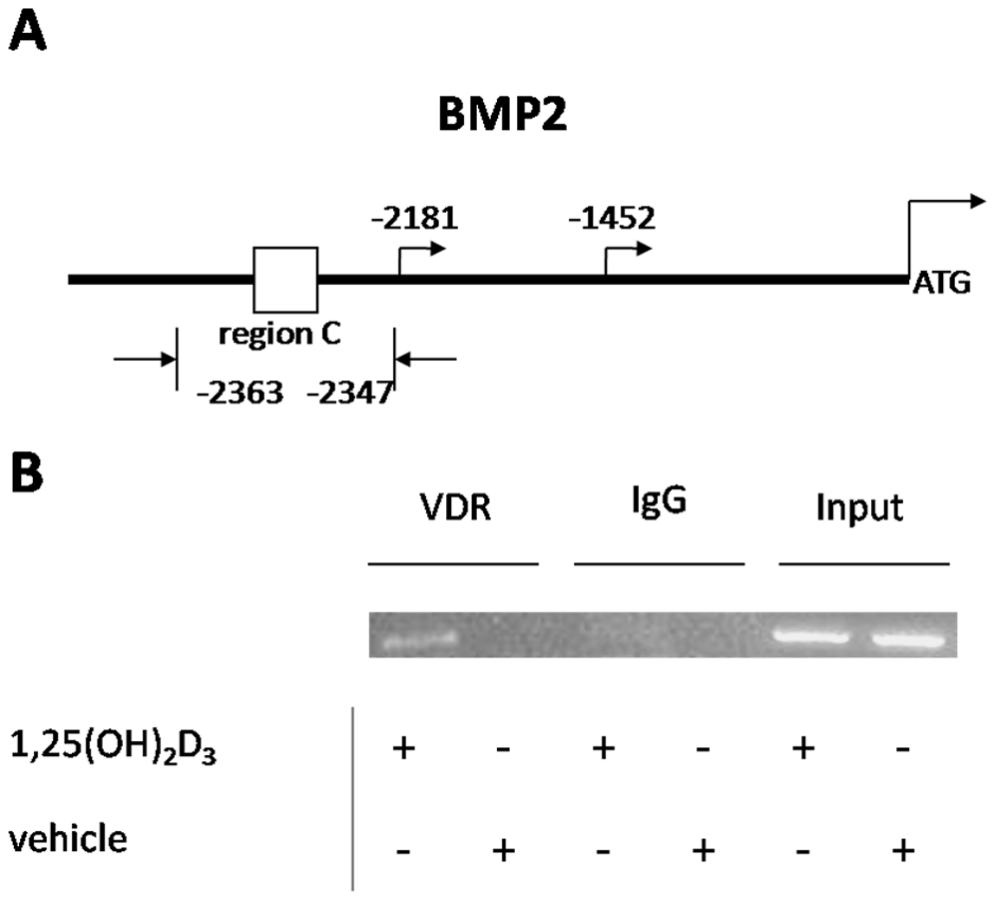
VDR binding to *BMP2* promoter region in vitro. ChIP assays were performed on UMR-106 cell extracts using anti-VDR antibody with or without 1,25(OH)_2_D_3_ administration. Normal IgG was used as a negative control. DNA was precipitated with either anti-VDR antibody or normal IgG. The primer pairs of putative VDREs in rat *BMP2* promoter region (as described in “Materials and Methods”) were used to amplify the ChIP DNA fragments. A. The site of *BMP2* promoter region C and the two corresponding promoters (−2181 and −1452) based on the conserved sequence data in mouse. B. The results showed that VDR bind to *BMP2* promoter region C.

We identified eight putative VDR binding sites in BMP2 promoter region, including positions and sequences (Table 1). Seven primer pairs were designed to match the putative binding sites. The ChIP DNA fragments from the UMR-106 cells were amplified via PCR. Significant binding of VDR to *BMP2* promoter region C was identified in cells incubated with 1,25(OH)_2_D_3_ (Figure. 3B). The other putative sites showed no binding to VDR (data not shown).

### DNA Methylation Reduces *BMP2* Gene Expression

**Figure 4:**
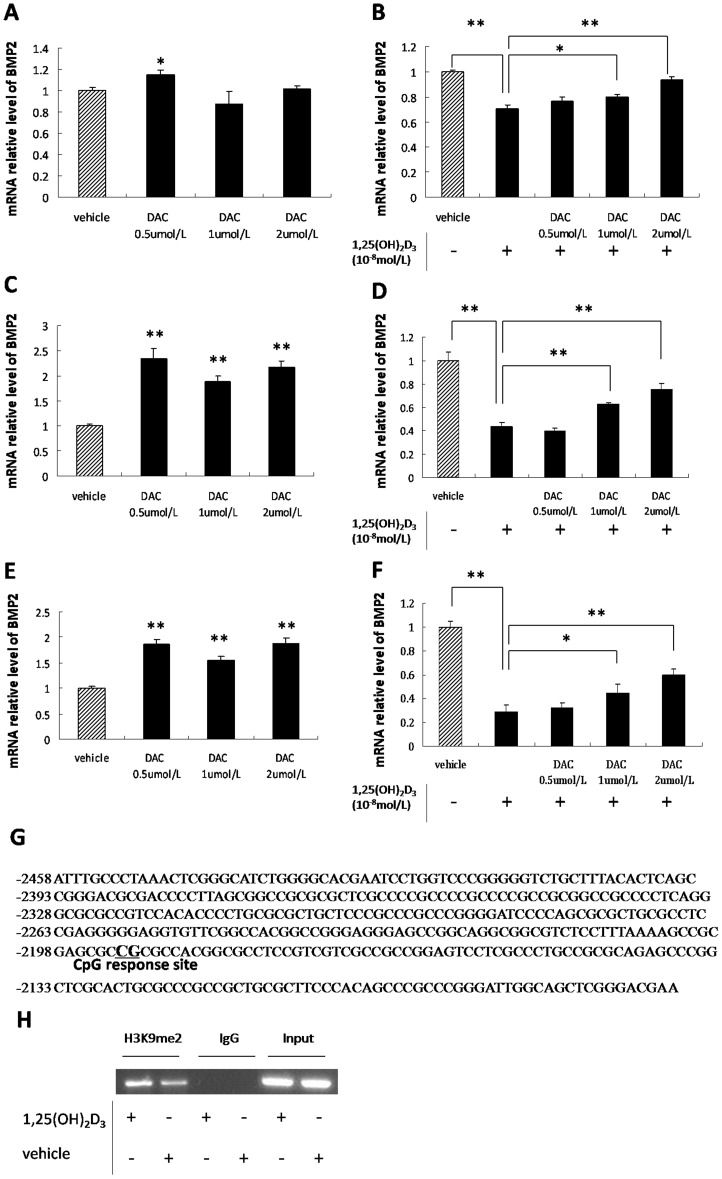
Activation of *BMP2* expression by DAC. A. qRT-PCR of *BMP2* expression in BMSCs from SD rats treated with varying concentration of DAC. B. qRT-PCR of *BMP2* expression in BMSCs from SD rats treated with varying concentrations of DAC followed by 1,25(OH)_2_D_3._ C. qRT-PCR of *BMP2* expression in BMSCs from GHS rats treated with varying concentrations of DAC. D. qRT-PCR of *BMP2* expression in BMSCs from GHS rats treated with varying concentrations of DAC followed by incubation with 1,25(OH)_2_D_3_. E. qRT-PCR of *BMP2* expression in UMR-106 cells treated with varying concentrations of DAC. F. qRT-PCR of *BMP2* expression in UMR-106 cells treated with varying concentrations of DAC followed by 1,25(OH)_2_D_3_. G. Bisulfate sequencing of *BMP2* promoter CpG island methylation in UMR-106 cells with 1,25(OH)_2_D_3_ administration and the CpG response site. The sequence is numbered relative to the translational start site. H. Antibody against H3K9me2 was used to do ChIP assay on chromatin extracted from UMR-106 cells which were incubated with 1,25(OH)_2_D_3_ or vehicle. The normal IgG was used as a negative control. Primer pairs designed for *BMP2* promoter region C were used in the PCR. (*, P<0.05; **,P<0.01).

BMSCs from SD and GHS rats were incubated with DAC, a DNA methyltransferase inhibitor. 0.5 uM DAC up-regulated *BMP2* expression in BMSCs from SD rats (Figure. 4A). Consistent up-regulation of *BMP2* expression occurred in BMSCs from GHS rats following DAC administration (Figure. 4C). When incubated with 1,25(OH)_2_D_3_, higher concentrations (1.0 and 2.0 µM) of DAC induced *BMP2* expression in BMSCs from both SD and GHS rats (Figure. 4B.D). The role of DNA methylation in regulation of *BMP2* expression was also explored in UMR-106 cells. In the presence of DAC, *BMP2* expression is up-regulated in UMR-106 cells. However, despite using a range of DAC concentrations, no significant difference of *BMP2* expression was detected (Figure. 4E). Incubation with both DAC and 1,25(OH)_2_D_3_ stimulated a concentration-dependent induction of *BMP2* expression (Figure. 4F).

Bisulfite pyrosequencing was used to test the potential role of DNA methylation in *BMP2* expression down-regulated following 1,25(OH)_2_D_3_ incubation. Bisulfite pyrosequencing was performed on the DNA fragment containing region C from UMR-106 cells incubated in the presence of 1,25(OH)_2_D_3_ or vehicle. Of the 57 CpGs sparsely spaced in this region of DNA, one was completely (100%, 25C/0T) methylated after incubation with 1,25(OH)_2_D_3_ (Figure. 4G). In the untreated control cell line, this CpG site was not methylated (Data not shown). The results indicate that 1,25(OH)_2_D_3_ represses *BMP2* expression through DNA methylation.

To clarify the role of histone methylation in 1,25(OH)_2_D_3_ induced regulation of the BMP2 gene, we examined the repressive histone mark H3K9me2 associated with the BMP2 promoter region using the ChIP assay. The result demonstrated that H3K9me2 was increased in the BMP2 promoter region C in the presence of 1,25(OH)_2_D_3_ in UMR-106 cells (Figure. 4H).

### Histone Modification is Associated with *BMP2* Expression

**Figure 5:**
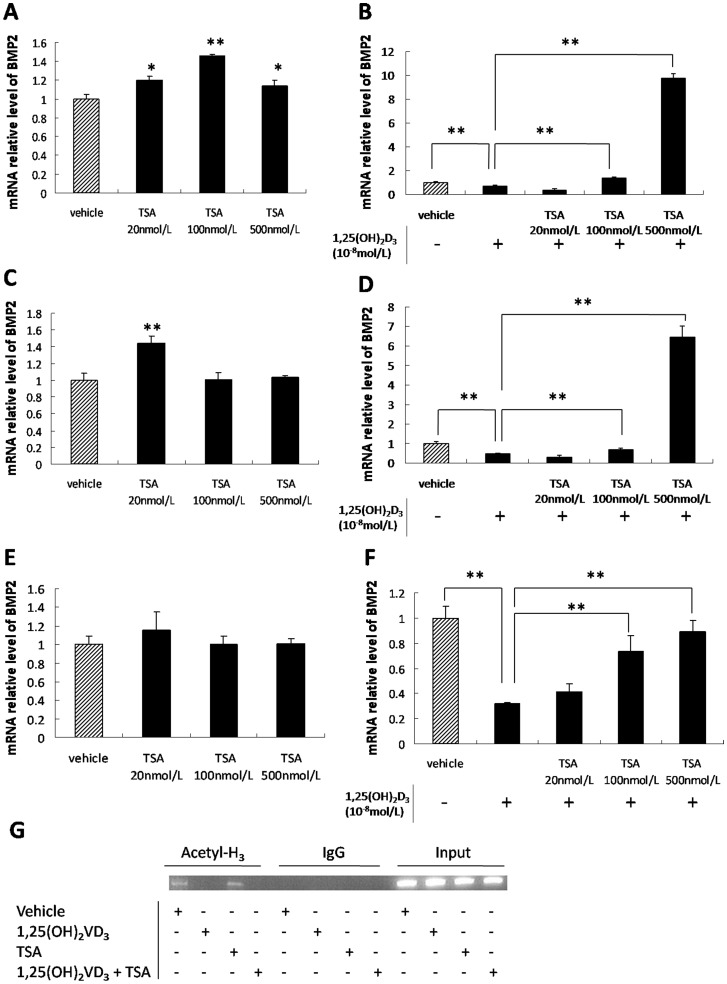
Histone modifications associated with *BMP2* expression. A. qRT-PCR of *BMP2* expression in BMSCs from SD rats treated with varying concentrations of TSA. B. qRT-PCR of *BMP2* expression in BMSCs from SD rats treated with varying concentrations of TSA followed by 1,25(OH)_2_D_3_. C. qRT-PCR of *BMP2* expression in BMSCs from GHS rats treated with varying concentrations of TSA. D. qRT-PCR of *BMP2* expression in BMSCs from GHS rats treated with varying concentrations of TSA followed by 1,25(OH)_2_D_3_. E. qRT-PCR of *BMP2* expression in UMR-106 cells treated with varying concentrations of TSA. F. qRT-PCR of *BMP2* expression in UMR-106 cells treated with varying concentrations of TSA followed by 1,25(OH)_2_D_3_. G. Antibody against acetyl-histone H3 was used to do ChIP assay on chromatin extracted from UMR-106 cells which were incubated with 1,25(OH)_2_D_3_ (together with or without TSA) or vehicle. The normal IgG was used as a negative control. Primer pairs designed for *BMP2* promoter region C were used in the PCR. (*, P<0.05; **, P<0.01).

TSA, a histone deacetylase inhibitor, up-regulated BMP2 expression in BMSCs from SD rats (Figure. 5A) and only 20 nM TSA increased *BMP2* expression in BMSCs from GHS rats (Figure. 5C). With 1,25(OH)_2_D_3_, both 100 and 500 nM TSA up-regulated *BMP2* expression in BMSCs from SD and GHS rats. The highest concentration increased *BMP2* expression by more than 10 times compared to 1,25(OH)_2_D_3_ administration alone (Figure. 5B.D).

Using UMR-106 cells, TSA elicited a dose-dependent induction of *BMP2* expression which was dependent upon 1,25(OH)_2_D_3_. A range of concentrations of TSA failed to change BMP2 expression in a dose-dependent manner (Figure. 5E). When UMR-106 cells were treated with either 100 or 500 nM TSA plus 1,25(OH)_2_D_3_, *BMP2* expression increased by 120% and 180%, respectively, compared to 1,25(OH)_2_D_3_ administration alone (Figure. 5F).

Using the ChIP assay, we found that 1,25(OH)_2_D_3_ decreased the acetylation of histone H3 obviously in the BMP2 promoter region C in UMR-106 cells, but this deacetylation was not reversed by the additional TSA treatment (Figure. 5G).

### VDR Represses *BMP2* Transcription through *BMP2* Promoter Region C

**Figure 6:**
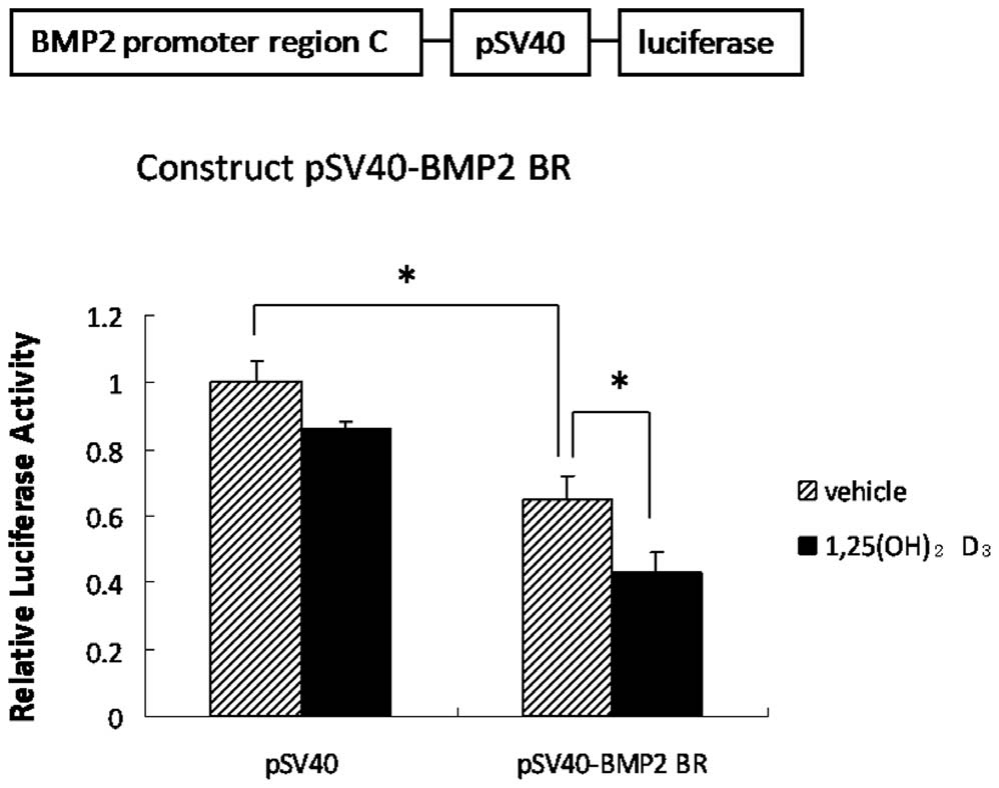
Effect of the VDR binding region in the BMP2 promoter. Luciferase activity in UMR-106 cells after 24 hr transfection with the construct pSV40-BMP2 BR-luciferase plasmid followed by 1,25(OH)_2_D_3_ or vehicle for an additional 24 hr is shown. The pGL3 Promoter Vector which contains an SV40 promoter upstream of the luciferase gene was used as a negative control. (*, P<0.05).

We amplified the VDR binding region involving rat *BMP2* promoter (region C) and constructed a pSV40-BMP2 BR-Luciferase plasmid. The construct was transfected into UMR-106 cells with the pCMV-βGal vector. Activity of the pSV40-BMP2 BR-Luciferase promoter was reduced by 40% following administration of 1,25(OH)_2_D_3_ (Figure. 6, P<0.05), whereas no difference was noted in the pSV40-Luciferase promoter activity (Figure. 6, P>0.05). These results indicate that VDR exerts its repressive effect through site-specific binding to the region C sequence.

## Discussion

Pathologic changes in calcium metabolism including increased calcium transport across intestine, bone and kidney which characterize IH are also found in GHS rats [7], [38], [39]. Bone mass is low in both human IH and GHS rats. In GHS rats, the low bone mineral density is due in part to both a decrease in trabecular volume and thickness with a resulting increase in fragility [7]. The cellular basis for the low bone mass in GHS rats is a defective function in both osteoclastic bone resorption and osteoblastic bone formation (unpublished data). In the present study, we found that *BMP2* expression is lower in BMSCs from GHS rats. BMP2 is a key osteogenic factor that is responsible for stimulating osteoblastic differentiation in BMSCs into mature osteoblasts which are then capable of synthesizing and secreting proteins that compose the bone matrix and ultimately stimulating bone growth. Thus, reduced *BMP2* expression in GHS rats is a major factor in reducing bone mass. We also demonstrated the higher VDR levels in GHS rat BMSCs. However, the interaction between VDR and *BMP2* is not fully known. In the present study, we found that *BMP2* gene expression is down-regulated by a direct action of 1,25(OH)_2_D_3_ when incubated in both BMSCs from SD and GHS rats and UMR-106 cells. The inhibitory action appears to be cell-specific, as *BMP2* expression was not changed by 1,25(OH)_2_D_3_ when incubated with two kidney cell lines HEK293 and RK3E (data not shown). So the lower bone mass in GHS rats may have been partly caused by the higher VDR levels which could repress BMP2 expression.

Most if not all of the biological actions of 1,25(OH)_2_D_3_ require binding to VDR followed by transcriptional regulation of a large number of genes that are responsible for the biologic actions associated with vitamin D [40], [41]. VDR regulates target gene expression by binding to specific DNA response elements [42]. In the present study, we identified a novel VDR binding site in the *BMP2* promoter region. According to the previous report [43,there are two conserved promoters initiating the BMP2 transcription in mouse. These regions are highly conserved between mouse and rat. By matching the rat sequence to that of mouse, we found that this novel VDR binding site is located at about 170180 bps upstream of the transcription start site of the distal promoter region (Figure. 3A). One other study has demonstrated two repressive regulatory elements located in the *BMP2* promoter and the upstream one localizes to the homologous position with *BMP2* promoter region C in our study [44]. Using the luciferase assay, the *BMP2* promoter region C mediates BMP2 gene repression following incubation with 1,25(OH)_2_D_3_. Therefore, the negative regulation of VDR on BMP2 gene expression is mediated by VDR binding to the novel VDR binding site within the rat *BMP2* promoter region.

Besides ligand-induced transcriptional repression by VDR, we observed co-regulator switching to be involved in the modulation of *BMP2* expression. DNA methylation is among those co-regulators with its actions primarily at CpG sites. Approximately 70% of gene promoter regions are located within CpG islands [45]. Methylation of CpG islands down-regulates gene expression during development and differentiation [46]–[48]. In the present study, there is a 5′CpG-rich island in BMP2 promoter region C which suggests a regulatory role of DNA methylation on *BMP2* gene expression. Pyrosequencing revealed an entirely methylated CpG in the *BMP2* promoter region C following 1,25(OH)_2_D_3_ administration in UMR-106 cells. This strongly suggests that 1,25(OH)_2_D_3_ complexes with the VDR and promote repression via methylation of the *BMP2* promoter. Moreover, the DNA methylation inhibitor up-regulated BMP2 expression and reversed the suppression by 1,25(OH)_2_D_3_ in both BMSCs from SD and GHS rats and UMR-106 cells. Furthermore, histone lysine methylation plays a key regulatory role in gene expression. Dimethylation of H3K9 is involved in transcriptional silencing and it is the critical mark for DNA methylation [49], [50]. In the current study, the ChIP assay demonstrated that 1,25(OH)_2_D_3_ increased the gene silence mark H3K9me2 in the *BMP2* promoter region C. Together, the results indicate that DNA methylation in the promoter region contributes, at least in part, to transcriptional down-regulation of *BMP2* expression by 1,25(OH)_2_D_3_.

In addition to VDR-mediated regulation of BMP2 expression through DNA methylation, histone acetylation may occur through a reversible modification controlled by histone acetylases (HATs) and histone deacetylases (HDACs) [51]. Acetylation of histones opens the chromatin structure and promotes transcription [45]. Site-specific acetylation or deacetylation in the promoter regions results in activation or repression of transcription, respectively [52]. In the present study, there was no consistent effect by histone deacetylation inhibitor alone on *BMP2* expression in either the BMSCs or UMR-106 cells. However, when the histone deacetylation inhibitor was added into the culture medium along with 1,25(OH)_2_D_3_, there was a concentration-dependent increase in *BMP2* expression. The ChIP assay demonstrated that 1,25(OH)_2_D_3_ reduced histone H3 acetylation specifically in the BMP2 promoter Region C. However, TSA treatment did not enhance histone H3 acetylation in the BMP2 promoter Region C in the present study. This might be because TSA has multiple effects on numerous gene expressions, which in turn directly or indirectly affects the chromatin modification of BMP2 gene. Of particular importance is the absence of acetylation of the histone H3 in Region C in the presence of 1,25(OH)_2_D_3._ The results strongly suggest that 1,25(OH)_2_D_3_ induces histone deacetylation and so contributes to *BMP2* repression.

In summary, the results presented suggest that 1,25(OH)_2_D_3_ down-regulates *BMP2* gene expression in osteoblast-like cells by activating a negative VDRE in the *BMP2* promoter. High levels of VDR mediate low bone mass in GHS rats partially through down-regulation of BMP2 expression.
