# Correction: Epigenetic Regulation of *BMP2* by 1,25-dihydroxyvitamin D_3_ through DNA Methylation and Histone Modification

**DOI:** 10.1371/annotation/1b1eb36e-60a4-4d28-bb3a-ee5a12a3d5d8

**Published:** 2013-09-13

**Authors:** Baisheng Fu, Hongwei Wang, Jinhua Wang, Ivana Barouhas, Wanqing Liu, Adam Shuboy, David A. Bushinsky, Dongsheng Zhou, Murray J. Favus

The sixth author (AS) was incorrectly omitted from the list of those authors who "Performed the experiment." 

